# Some ethylene biosynthesis and AP2/ERF genes reveal a specific pattern of expression during somatic embryogenesis in *Hevea brasiliensis*

**DOI:** 10.1186/1471-2229-12-244

**Published:** 2012-12-26

**Authors:** Piyanuch Piyatrakul, Riza-Arief Putranto, Florence Martin, Maryannick Rio, Florence Dessailly, Julie Leclercq, Jean-François Dufayard, Ludovic Lardet, Pascal Montoro

**Affiliations:** 1 CIRAD, UMR AGAP, F-34398, Montpellier, France; 2 Rubber Research Institute, Chatuchak, Bangkok, 10900, Thailand; 3 Indonesian Biotechnology Research Institute for Estate Crops, Bogor, Indonesia

**Keywords:** Gene expression, Plant hormone, Plant regeneration, Recalcitrant, Rubber, Signalling, Transcription factor

## Abstract

**Background:**

Ethylene production and signalling play an important role in somatic embryogenesis, especially for species that are recalcitrant in *in vitro* culture. The AP2/ERF superfamily has been identified and classified in *Hevea brasiliensis*. This superfamily includes the ERFs involved in response to ethylene. The relative transcript abundance of ethylene biosynthesis genes and of AP2/ERF genes was analysed during somatic embryogenesis for callus lines with different regeneration potential, in order to identify genes regulated during that process.

**Results:**

The analysis of relative transcript abundance was carried out by real-time RT-PCR for 142 genes. The transcripts of ERFs from group I, VII and VIII were abundant at all stages of the somatic embryogenesis process. Forty genetic expression markers for callus regeneration capacity were identified. Fourteen markers were found for proliferating calli and 35 markers for calli at the end of the embryogenesis induction phase. Sixteen markers discriminated between normal and abnormal embryos and, lastly, there were 36 markers of conversion into plantlets. A phylogenetic analysis comparing the sequences of the AP2 domains of *Hevea* and *Arabidopsis* genes enabled us to predict the function of 13 expression marker genes.

**Conclusions:**

This first characterization of the AP2/ERF superfamily in *Hevea* revealed dramatic regulation of the expression of AP2/ERF genes during the somatic embryogenesis process. The gene expression markers of proliferating callus capacity to regenerate plants by somatic embryogenesis should make it possible to predict callus lines suitable to be used for multiplication. Further functional characterization of these markers opens up prospects for discovering specific AP2/ERF functions in the *Hevea* species for which somatic embryogenesis is difficult.

## Background

Understanding the molecular mechanisms controlling somatic embryogenesis is crucial, be it biologically or for applications. Indeed, propagating cultivated plants by somatic embryogenesis still remains limited by the efficiency of the procedures in some recalcitrant species or genotypes. Genetic predeterminism of recalcitrance in some woody and herbaceous perennial species has been linked to their response to wounding and their phenolic compound content [1]. The production of ethylene, carbon dioxide and free radicals lies behind the induction of cell defence mechanisms, leading to the oxidation of phenol compounds, the breakdown of cell walls and membrane peroxidation [2]. Adding growth regulators, and the stress induced by *in vitro* culture, play a leading role in such mechanisms [3]. These phenomena result in the differentiation of undifferentiated cells and in tissue browning, which lead to a loss in embryogenic capacity [2,4].

The negative effect of ethylene on somatic embryogenesis has been known for a long time [5]. Some super-embryogenic explant cultures of *Medicago truncatula* revealed the repression of numerous genes, including those involved in ethylene biosynthesis and signalling [6]. In alfalfa, loss of embryogenic capacity following thidiazuron application is linked to the induction of an ethylene biosynthesis gene [7]. Ethylene also induces some stress factors conducive to the acquisition of somatic embryogenesis capacities [8,9], and embryo maturation [10]. Abscisic acid and methyl jasmonate are regulators of ethylene biosynthesis during somatic embryogenesis in *Medicago sativa*[11]. Some members of the ETHYLENE RESPONSE FACTOR (ERF) family are involved in response to *in vitro* stress and in the regulation of developmental processes. In *Medicago truncatula*, the *SOMATIC EMBRYO RELATED FACTOR 1* gene (*MtSERF1*) is induced by ethylene and may act under the influence of WUSCHEL (WUS), whose fixation sites are found on the *SERF1* gene promoter [12]. ENHANCER OF SHOOT REGENERATION1 (ESR1) of *Arabidopsis* is induced by cytokinins to regulate the start of shoot regeneration [13]. DORNROSCHEN (DRN)/ESR1 plays a role in meristem and organ development and consequently in shoot regeneration [14]. ESR2 expression confers “cytokinin-independent shoot regeneration” through transcriptional regulation of the *CUP-SHAPED COTYLEDON 1* gene (*CUC1*) [15]. When interacting with other transcription factors, the induction of CALLUS EXPRESSING FACTOR1 (CEF1) through stress would appear to disrupt auxin/cytokinin homeostasis in *Nicotiana tabacum*. Of the numerous other transcription factors involved in embryogenesis and organ development ([16] for a review), several members of the APETALA2 family (AP2) play a major role [17]. Like ERFs, this family belongs to the AP2/ERF superfamily. For example, BABY BOOM (BBM) is known for its role in cell proliferation and morphogenesis during embryogenesis [18]. AINTEGUMENTA (ANT) is involved in ovule development and in the growth of floral organs [19]. WRINKLED (WRl1) is involved in regulating storage metabolism in seeds [20,21]. The ANT subfamily includes several members called ANT-like genes or AILs. The initiation of post-embryonic shoot organs takes place in the shoot apical meristem, also involving several members of the AP2 family (ANT, AIL6/PLT3 and AIL7/PLT7) [22].

*Hevea brasiliensis* is a particularly difficult species which has led to numerous micropropagation studies [23]. This cross-fertilizing species is cloned by budding in the absence of any other efficient vegetative propagation techniques for own-rooted plants. Two somatic embryogenesis methods have been developed for *Hevea* ([23-25] for a review). The first is somatic embryogenesis on primary callus (SEP) obtained from fragments of the internal integument of immature seeds or anthers [26-28]. This method is effective for around twenty *Hevea* clones following numerous culture medium and atmosphere studies [25,29]. However, primary calli are subject to browning due to high ethylene and carbon dioxide release, which leads to a low callus multiplication rate [30]. *In vitro* plantlets produced by SEP are usually of good quality with better growth and latex production than budded clones [31]. A second method was developed from friable callus maintained over the long term with a view to large-scale multiplication. This method has evolved with the use of fragments of embryos derived from SEP in order to rapidly establish embryogenic friable callus lines [29,32]. Lastly, cryopreservation of friable calli has been incorporated into the process to limit tissue proliferation and, thereby, the risks linked to somaclonal variation [33]. However, this indirect secondary somatic embryogenesis process is restricted to just a few clones and the embryo and plant regeneration capacity is variable. The absence of plant regeneration potential in some friable callus lines has been linked to early vacuolization of cells in the embryogenic globules [34]. Some transcriptional changes have also been reported for certain *Hevea* callus lines with different embryogenic potentials [34].

A study on the role of ethylene in stimulating latex production led to the characterization of ethylene biosynthesis and signalling genes and more recently to the identification of the different members of the AP2/ERF superfamily [35-38]. The AP2/ERF superfamily contains 173 members, of which 142 are classed in families and groups [35]. The AP2 family contains 20 genes organized in two subfamilies (8 ANT and 12 AP2 genes). The ERF family is divided into 10 groups comprising a total of 115 genes. Lastly, there remains the RAV family with four genes and the soloists with 3 genes. Based on this knowledge, our study set out to gain a clearer understanding of the regulation of ethylene biosynthesis and signalling genes, including ERFs, along with the members of the AP2 and RAV families during the somatic embryogenesis process in *Hevea*. The relative transcript abundance analysis for these genes was carried out by real-time RT-PCR on friable callus lines with different embryogenic potential [33], on normal and abnormal somatic embryos, and on different young plant tissues. The gene expression profiles showed that several genes were markers of embryogenic potential for the lines in proliferating calli and in calli induced by embryogenesis, and also markers of somatic embryo quality. Prediction of the function of those marker genes provides a dynamic understanding of the somatic embryogenesis process.

## Results

### Morphogenetic potential of callus lines with different somatic embryogenesis capacities

**Figure 1:**
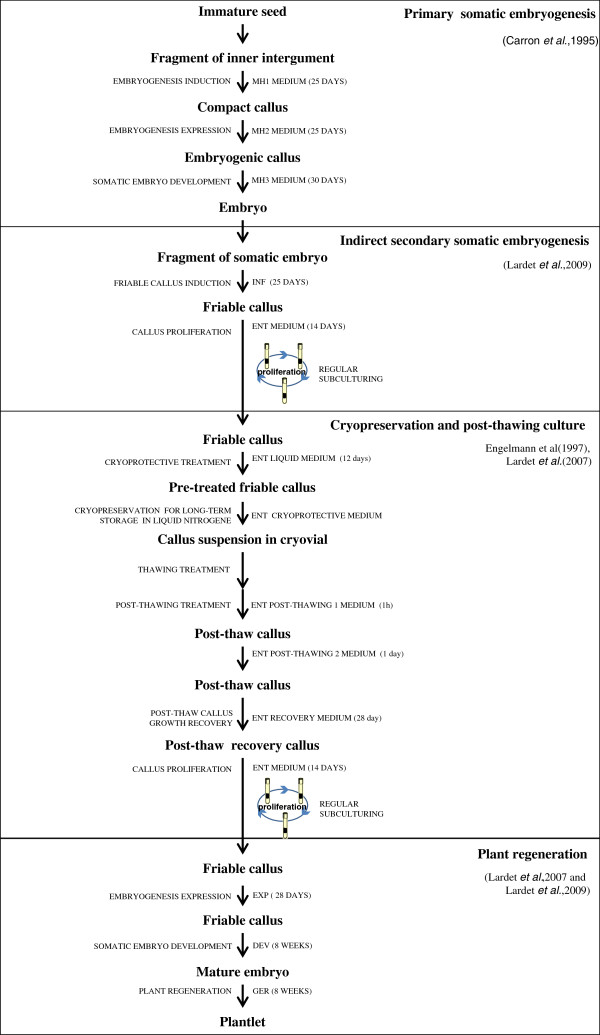
**Plant regeneration process for *Hevea brasiliensis.*** Primary somatic embryogenesis using the inner integument of immature seeds, indirect secondary somatic embryogenesis, cryopreservation and plant regeneration methods have been described in several papers [24,29,33,39].

**Figure 2:**
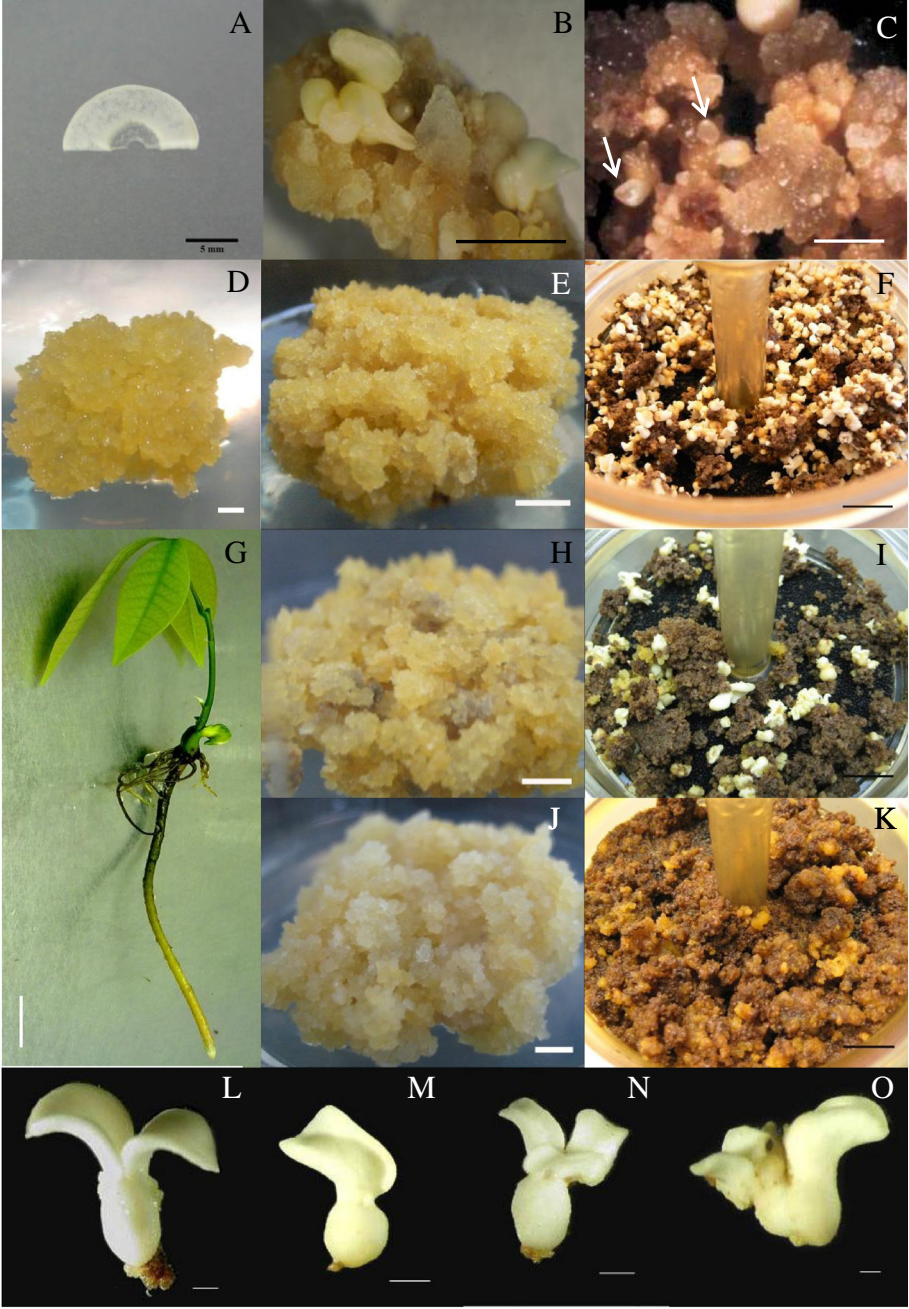
**Morphology of callus with different embryogenic capacities and somatic embryos.** (**A**) Inner integument of immature seed (scale bar = 5 mm). (**B**) Compact callus on somatic embryo development medium (MH3) from primary somatic embryogenesis (bar = 5 mm). (**C**) Embryogenic callus bearing pro-embryo structures (white arrow) on somatic embryo development medium (DEV) (bar = 5 mm). (**D**) Regenerant line callus on embryogenesis induction medium (ENT) (bar = 1 mm). Calli were from three types of lines on somatic embryogenesis expression medium (EXP): (**E**) regenerant line callus, (**H**) embryogenic and non-regenerant line and (**J**) non-embryogenic line on somatic embryogenesis expression medium (EXP) (bar = 5 mm). Friable callus on embryo development medium (DEV): (**F**) from regenerant line callus, (**I**) embryogenic and non-regenerant line and (**K**) non-embryogenic line (bar = 10 mm). Various types of somatic embryo: (**L**) normal cotyledonated embryo, and (**M, N, O**) abnormal somatic embryos (bar = 1 mm). (**G**) Plantlet from a normal somatic embryo (bar = 10 mm).

The secondary indirect somatic embryogenesis process is described in Figure 1 (cf. Materials and Methods). Primary somatic embryogenesis is induced from inner integument of immature seed as maternal tissue (Figure 2A). Compact callus-bearing mature somatic embryos were obtained after three subcultures (Figure 2B). Friable callus lines were established from fragments of somatic embryo. The proliferating friable calli grown on the ENT medium were fairly comparable whatever their embryogenic capacity (Figure 2D). After four weeks’ culture on EXP, the calli of the non-embryogenic (Figure 2J) and embryogenic (Figure 2H) lines were more hydric and whitish than those of the regenerant line (Figure 2E). Callus growth was slowed during embryo regeneration in the RITA culture system on DEV medium. The calli turned brown in all the lines (Figure 2F, I and K). Browning occurred to the benefit of embryo formation for embryogenic lines (Figure 2F and I). The normal embryos had an embryonic body and two well-developed cotyledons (Figure 2L). The abnormal types of embryos were most numerous: for example, some embryos with a single cotyledon (Figure 2M), or with malformed cotyledons (Figure 2N), and a double embryonic body (Figure 2O) were found. Plantlets derived from normal embryos developed a taproot and a lateral root system, and a stem with leaves within a month (Figure 2G).

The morphogenetic capacities of three friable callus lines were tested up to somatic embryo conversion into plantlets (Table 1). The non-embryogenic line CI04115 (NE) did not produce any somatic embryos. Line CI04106, called embryogenic (E), produced 19.5 somatic embryos per gram of callus, most of which were abnormal (93.54%). Only the regenerant line CI07060 (R) produced a large number of total somatic embryos (590 per gram of callus). The proportion of abnormal embryos was very high for the embryogenic and regenerant lines, at 93.54% and 82.71% respectively. Out of 102 normal embryos derived from the regenerant line transferred to germination medium, 53 developed into plantlets.

**Table 1:** Morphogenetic capacities of three callus lines

**Embryogenic capacity**	**Line**	**Embryo**					**Plantlet from normal embryo**	
		**Total**	**Normal**		**Abnormal**			
		**(No/g**^**-1**^ **cal)**	**(No/g**^**-1**^ **cal)**	**(%)**	**(No/g**^**-1**^ **cal)**	**(%)**	**(No/g**^**-1**^ **cal)**	**(%)**
Non-embryogenic	CI04115	0.00 ± 0.00 ^a^	0.00 ± 0.00 ^a^	-	0.00 ± 0.00 ^a^	-	-	-
Embryogenic	CI04106	19.50 ± 19.13 ^a^	0.65 ± 1.17 ^a^	3.33	18.24 ± 17.37 ^a^	93.54	0.00 ± 0.00 ^a^	-
Regenerant	CI07060	591.00 ± 52.66 ^b^	102.20 ± 57.75 ^b^	17.28	488.80 ± 65.02 ^b^	82.71	53.40 ± 36.81 ^b^	52.25

The non-embryogenic line (CI 04115) cannot regenerate any embryos, the embryogenic line (CI 04106) can only produce a few embryos but cannot regenerate any plantlets, and the regenerant line (CI 07 060) can produce a large number of embryos and regenerate plantlets.

### Identification of 14 marker genes of somatic embryogenesis capacities during callus proliferation

**Figure 3:**
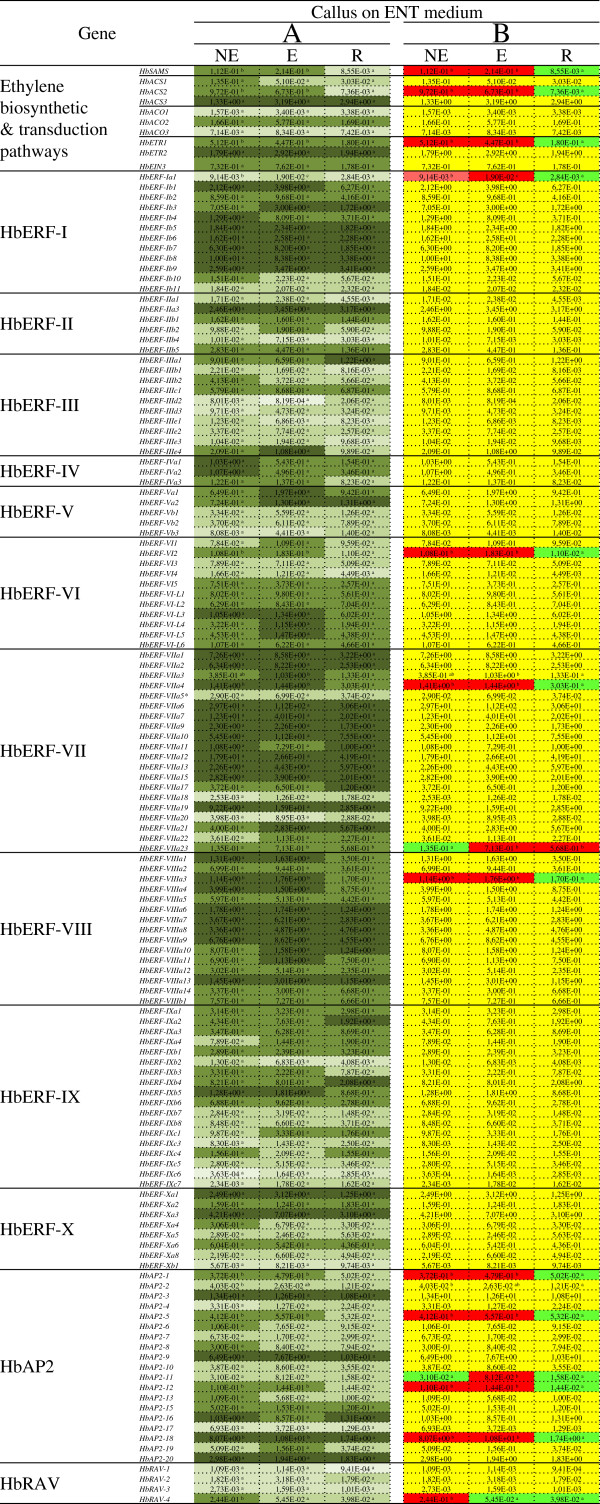
**Expression profile of 142 genes involved in the ethylene biosynthesis and signalling pathways for callus with different embryogenic capacities grown for 2 weeks on embryogenesis induction medium (ENT).** Calli were from three types of lines: (NE) non-embryogenic line (CI04115), (E) embryogenic and non-regenerant line (CI04106), and (R) regenerant line (CI07060). The relative transcript abundances were measured by real-time RT-PCR. Values are the means of the relative transcript abundances of three biological replicates. (Figure 3**A**) Heat map representation of the expression profile was used for values ranging as follows ≥ 1, 10^-1^, 10^-2^, 10^-3^ and ≤ 10^−4^ from dark to light green. (Figure 3**B**) Values of relative transcript abundance in callus of the various lines were analysed with XLSTAT software after log transformation. The statistical analysis was performed with an ANOVA followed by the Student Newman–Keuls test. Values with significantly high relative transcript abundances shown in red and significantly low relative transcript abundances shown in green. The non-significant genes are shown in yellow.

The relative transcript abundance for ethylene biosynthesis and signalling genes, and for the AP2 and RAV families, was analysed in proliferating calli on ENT medium (Figure 3). Some high levels were found for several genes in each family of genes, except for the RAVs (Figure 3A). The ERFs from groups I, VII and VIII amounted to 8, 15 and 10 genes respectively with levels over 1 in relative value compared to the transcript abundance of the internal control RH2b. The comparison between lines with different embryogenic potential revealed that 14 genes were differentially expressed (Figure 3B). The relative transcript abundance was lower in the calli of the R line than in the E and NE lines for 11 genes, of which three were involved in ethylene biosynthesis and perception (*HbSAMS, HbACS2, HbETR1*), four were from the ERF family (*HbERF-Ia1, HbERF-VI2, HbERF-VIIa4, HbERF-VIIIa3*), and 4 were from the AP2 family (*HbAP2-1, HbAP2-5, HbAP2-12, HbAP2-18*). The transcripts of the *HbAP2-11* gene were greatly accumulated in the embryogenic line. Lastly, the transcripts of the *HbERF-VIIa23* gene were less abundant in the non-embryogenic line, while, conversely, those of the *HbRAV-4* gene were accumulated more in the calli of that line.

### Identification of 35 marker genes of somatic embryogenesis capacities during somatic embryogenesis expression/induction

**Figure 4:**
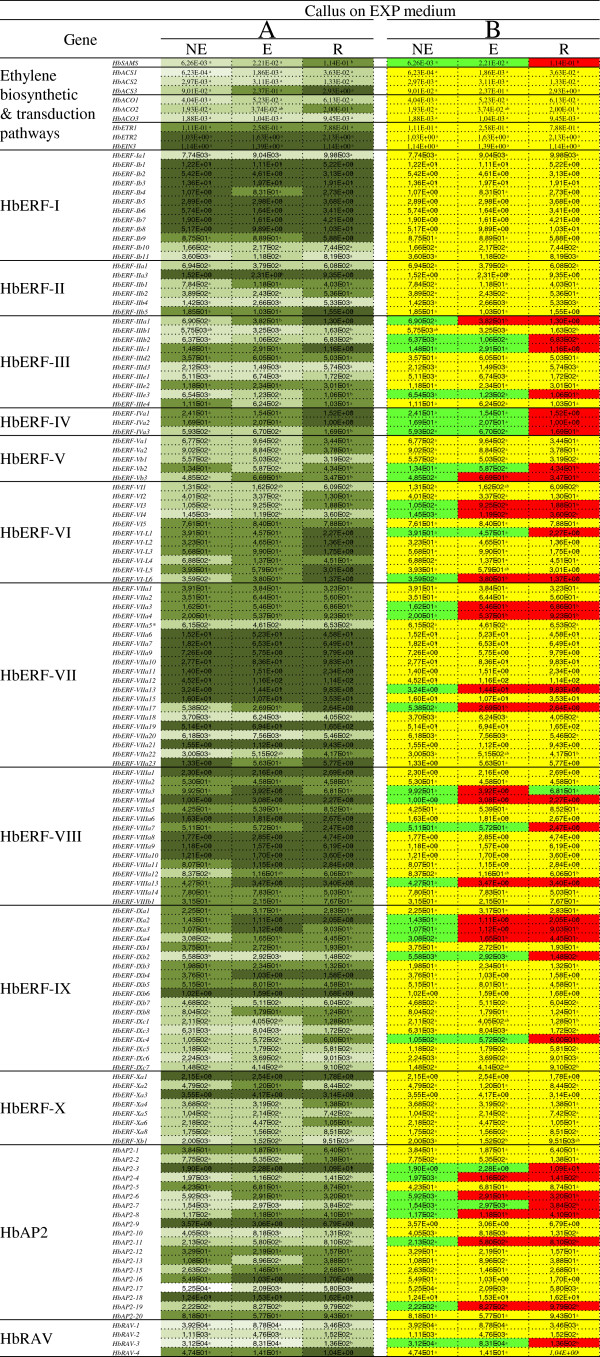
**Expression profile of 142 genes involved in the ethylene biosynthesis and signalling pathways for callus with different embryogenic capacities grown for 4 weeks on somatic embryogenesis expression medium (EXP).** Calli were from three types of lines: (NE) non-embryogenic line (CI04115), (E) embryogenic and non-regenerant line (CI04106), and (R) regenerant line (CI07060). The relative transcript abundances were measured by real-time RT-PCR. Values are the means of the relative transcript abundance of three biological replicates. (Figure 4**A**) Heat map representation of the expression profile was used for values ranging as follows ≥ 1, 10^-1^, 10^-2^, 10^-3^ and ≤ 10^−4^ from dark to light green. (Figure 4**B**) Values of relative transcript abundance in callus of the various lines were analysed with XLSTAT software after log transformation. The statistical analysis was performed with an ANOVA followed by the Student Newman–Keuls test. Values with significantly high relative transcript abundances shown in red and significantly low relative transcript abundances shown in green. The non-significant genes are shown in yellow.

The calli transferred to a somatic embryogenesis induction medium also strongly expressed a large number of the genes studied for virtually all the families and groups, except for the ERF-V group (Figure 4A). As previously, most of the members of groups I (9 genes), VII (12 genes) and VIII (10 genes) of the ERF family had a high transcript level. The differential expression between calli of the different lines was significant for 35 genes (Figure 4B). Of those genes, 15 had higher transcript accumulation in the regenerant line than in the NE and E lines: 1 ethylene biosynthesis gene (*HbSAMS*), 11 genes of the ERF family (*HbERF-IIIb2, HbERF-IIIc1, HbERF-IIIe3, HbERF-IVa1,HbERF-IVa2, HbERF-IVa3, HbERF-Vb2, HbERF-VI-L1, HbERF-VIIIa7, HbERF-IXb2, HbERF-IXc4*), 2 genes of the AP2 family (*HbAP2-3, HbAP2-7*) and 1 gene of the RAV family (*HbRAV-3*). Transcripts of the *HbERF-VIIIa3* gene were accumulated in the calli of the embryogenic line compared to the other two lines. Lastly, 19 genes comprising 14 ERFs (*HbERF-IIIa1, HbERF-Vb3, HbERF-VI3, HbERF-VI4, HbERF-VI-L6, HbERF-VIIa3, HbERF-VIIa4, HbERF-VIIa13, HbERF-VIIa17, HbERF-VIIIa4, HbERF-VIIIa13, HbERF-IXa2, HbERF-IXa3, HbERF-IXa4*) and 5 AP2s (*HbAP2-4, HbAP2-6, HbAP2-8, HbAP2-11, HbAP2-19*) had a lower transcript level in the non-embryogenic line than in the others.

### Sixteen genes were differentially expressed in normal and abnormal somatic embryos

**Figure 5:**
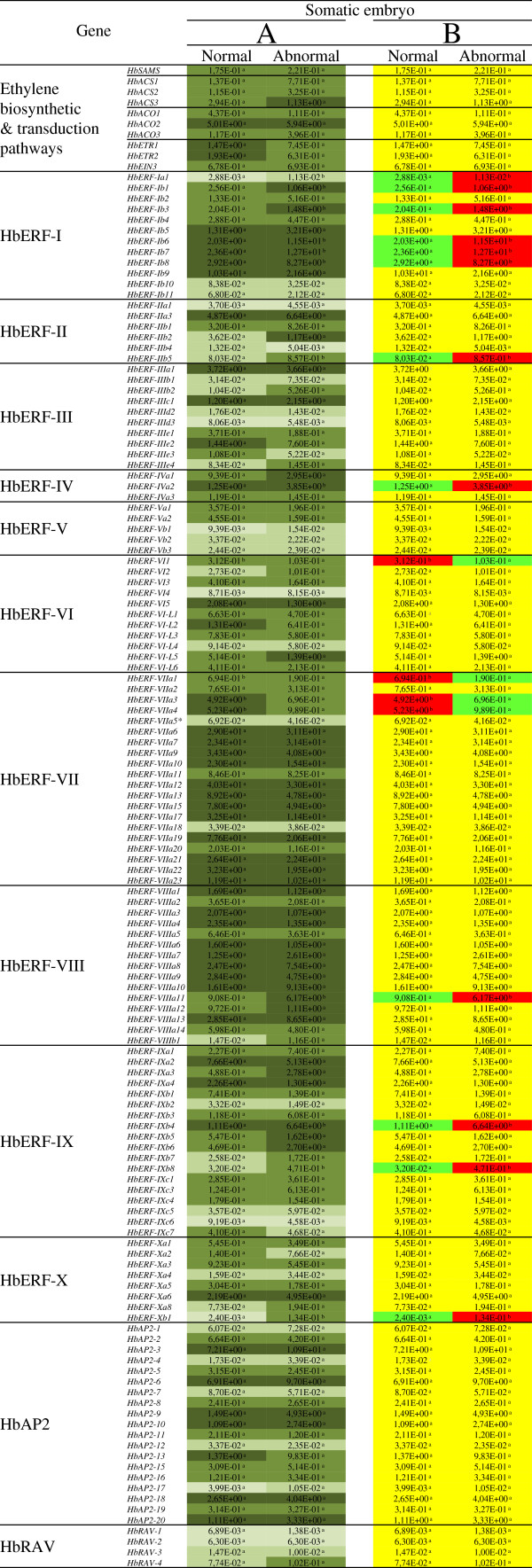
**Expression profile of 142 genes involved in the ethylene biosynthesis and signalling pathways for normal and abnormal embryos from regenerant line (CI07060) callus on embryo development medium (DEV).** The relative transcript abundances were measured by real-time RT-PCR. Values are the means of the relative transcript abundance of three biological replicates. (Figure 5**A**) Heat map representation of the expression profile was used for values ranging as follows ≥ 1, 10^-1^, 10^-2^, 10^-3^and ≤ 10^−4^ from dark to light green. (Figure 5**B**) Values of relative transcript abundance in normal and abnormal embryos were analysed with XLSTAT software after log transformation. The statistical analysis was performed with an ANOVA followed by the Student Newman–Keuls test. Values with significantly high relative transcript abundances shown in red and significantly low relative transcript abundances shown in green. The non-significant genes are shown in yellow.

The normal and abnormal somatic embryos produced by the regenerant line revealed a differential expression profile solely for certain members of the ERF family (Figure 5). The gene transcripts belonging to each of the families and groups were highly accumulated, except for the RAV family (Figure 5A). Most of the members of the ERF-I, ERF-VII and ERF-VIII groups were highly expressed in the two types of embryos. The transcript abundance was lower in the normal embryos than in the abnormal embryos for 12 genes belonging to the ERF family (*HbERF-Ia1, HbERF-Ib1, HbERF-Ib3, HbERF-Ib6, HbERF-Ib7, HbERF-Ib8, HbERF-IIb5, HbERF-IVa2, HbERF-VIIIa11, HbERF-IXb4, HbERF-IXb8, HbERF-Xb1*) (Figure 5B)*.* Conversely, the transcripts of 4 genes were accumulated in the normal embryos (*HbERF-VI1, HbERF-VIIa1, HbERF-VIIa3, HbERF-VIIa4*).

### Evolution of transcript abundance during the process of somatic embryogenesis and conversion into plantlets

**Figure 6:**
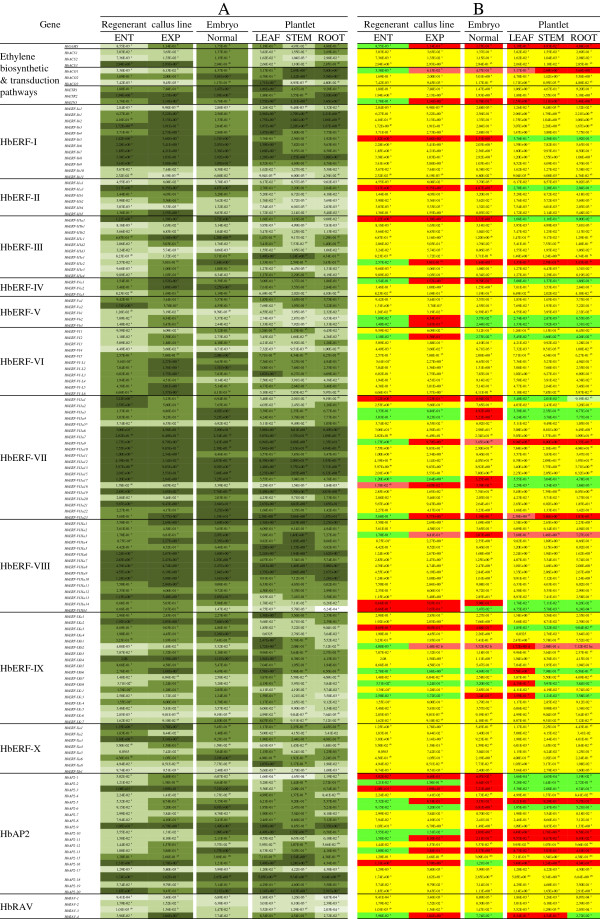
**Expression profile of 142 genes involved in the ethylene biosynthesis and signalling pathways for regenerant line (CI07060) callus on embryogenesis induction medium (ENT) and somatic embryogenesis expression medium (EXP), embryos and 3 plantlet organs: (L) Leaf, (S) Stem, (R) Root.** The relative transcript abundances were measured by real-time RT-PCR. Values are the means of the relative transcript abundance of three biological replicates. (Figure 6**A**) Heat map representation of the expression profile was used for values ranging as follows ≥ 1, 10^-1^, 10^-2^, 10^-3^ and ≤ 10^−4^ from dark to light green. (Figure 6**B**) Values of relative transcript abundance were analysed with XLSTAT software after log transformation. The statistical analysis was performed with an ANOVA followed by the Student Newman–Keuls test. Values with significantly high relative transcript abundances shown in red and significantly low relative transcript abundances shown in green. The non-significant genes are shown in yellow.

An overall analysis of the somatic embryogenesis process for the regenerant line using proliferating callus up to the conversion of normal embryos into plantlets showed that the expression of numerous genes was highly modulated (Figure 6). The transcripts of numerous ethylene biosynthesis and transduction genes, and some ERFs from groups I, VII and VIII and of the AP2 family, were highly accumulated (Figure 6A). Thirty-six genes from all the families or groups revealed differential accumulation during the embryogenesis process, except for the ERFs from group X (Figure 6B). Several gene expression profiles were observed. Eleven markers were activated right from the somatic embryogenesis induction phase (*HbSAMS, HbACO1, HbEIN3, HbERF-IIIe2, HbERF-VIIa9, HbERF-VIIa23, HbERF-VIIIa3, HbERF-IXb2, HbAP2-5, HbAP2-11, HbAP2-13*). The transcripts of eight genes (*HbERF-Ib5, HbERF-IIa3, HbERF-IIIa1, HbERF-VIIa1, HbERF-VIIIa14, HbERF-IXa3, HbAP2-1, HbAP2-3*) were very abundant in embryogenic tissues, whereas they were significantly less abundant in the tissues of the plantlet. A transient accumulation was seen for two genes (HbERF-Vb2, HbERF-Vb3) in calli induced in embryogenesis on EXP medium, for six genes (HbERF-VIIa3, HbERF-VIIa4, HbERF-VIIa17, HbERF-VIIIa3, HbAP2-2, HbAP2-6) in somatic embryos and for two genes (HbERF-IVa1, HbERF-VIIa18) in both calli on EXP medium and in embryos. The transcripts of three genes (HbERF-IXb2, HbERF-IXb6, HbERF-IXb8) were preferentially accumulated in leaves and, in the case of HbERF-IXc3, in both somatic embryos and leaves. Lastly, the relative transcript abundance was high in all tissues except in embryos, where it decreased significantly for the *HbAP2-16* gene, and except in calli, embryos and roots for *HbRAV4*.

### Identification of putative functions for somatic embryogenesis marker genes

Of the fifty-six genes differentially expressed during the somatic embryogenesis process, forty were regulated in the same way when the regenerant line was compared with the other two non-embryogenic or embryogenic lines, and when the normal embryos were compared with the abnormal embryos (Table 2). The ratios between the relative transcript abundance of the regenerant line and the other two lines showed that the markers tended to be under-expressed in the proliferating calli of the regenerant line (from 0.01 to 0.4) and over-expressed in the calli induced in embryogenesis on EXP medium (from 2 to 43 times). Of the sixteen embryo expression markers, twelve were under-expressed and four were over-expressed.

**Table 2:** **Identification of putative functions for somatic embryogenesis marker genes based on the reciprocical best hit analysis (BLASTX) using full cDNA sequences, and then co-orthology relationships, inferred by phylogeny, using the deduced amino acid sequences of the AP2 domain for each gene (see Additional file**1**: Figure S1, Additional file**2**: Figure S2, Additional file**3**: Figure S3, Additional file**4**: Figure S4, Additional file**5**: figure S5 and Additional file**6**: Figure S6)**

**Gene**	**Callus on ENT**		**Callus on EXP**		**Embryo**	**Reciprocal best hit analysis**				**Phylogenetic analysis**			
	**R/NE**	**R/E**	**R/NE**	**R/E**	**Nor/Ab**	**Homology gene**	**Putative function**	**Species**	**Reference**	**Orthologous gene**	**Accession No**	**Putative Function**	**Reference**
*HbSAMS*	0.08	0.04	18.20	5.16		SAMS	Precursor for ethylene biosynthesis	*Eucalyptus*	[40,41]	.			
*HbACS2*	0.01	0.01				ACS	ACS synthase	*Ricinus*	[41,42]	.			
*HbETR1*	0.35	0.40				HbETR	Signal transduction	*Hevea*	[41,42]	.			
*HbERF-Ia1*	0.31	0.15			0.26	DREB	Freezing & dehydration tolerance	*Glycine*	[43-45]	ERF53	At2g20880	Regulates drought-responsive gene expression	[46]
*HbERF-Ib1*					0.24	DREB	Freezing & dehydration tolerance	*Populus*	[43-45]	.	At4g39780	Unknown	
*HbERF-Ib3*					0.14	DREB	Freezing & dehydration tolerance	*Populus*	[43-45]	.	At4g39780	Unknown	
*HbERF-Ib6*					0.18	DREB1p	Dwarfed phenotypes,freezing & dehydration tolerance	*Hevea*	[44]	.			
*HbERF-Ib7*					0.19	DREB1p	Dwarfed phenotypes,freezing & dehydration tolerance	*Hevea*	[44]	.			
*HbERF-Ib8*					0.35	DREB1p	Dwarfed phenotypes,freezing & dehydration tolerance	*Hevea*	[44]	.			
*HbERF-IIb5*					0.09	TINY	Affects plant height, hypocotyl elongation, and fertility	*Populus*	[47,48]	.	At1g44830	Unknown	
*HbERF-IIIa1*			18.80	3.40		TINY	Affects plant height, hypocotyl elongation, and fertility	*Populus*	[47,48]	.	At1g01250	Unknown	
*HbERF-IIIb2*			10.70	6.44		ERF025	Control of ethylene-responsive transcription genes	*Glycine*	[49]	.	At1g63040	Unknown	
*HbERF-IIIc1*			7.84	3.99		CRT/DRE	Heat stress tolerance	*Hevea*	[50,51]	CBF3/DREB1A	At4g25480	Response to low temperature, abscisic acid	[52]
										CBF2/DREB1C	At4g25470	Response to low temperature, abscisic acid,	[53]
										CBF1/DREB1B	At4g25490	Response to low temperature, abscisic acid	[52]
										CBF4/DREB1D	At5g51990	Response to drought stress and abscisic acid	[54]
										DDF2	At1g63030	Regulates in GA biosynthesis and stress tolerance	[55]
										DDF1	At1g12610	Regulates in GA biosynthesis and stress tolerance	[55]
*HbERF-IIIe3*			16.20	8.62		TINY	Affects plant height, hypocotyl elongation, and fertility	*Populus*	[47,48]	TINY	At5g25810	Suppresses cell proliferation and exhibits pleiotropic effects	[47]
*HbERF-IVa1*			6.31	9.87		DREB2	Enhances drought stress tolerance	*Populus*	[50,51]	DREB2A	At5g05410	Drought-responsive gene expression	[50]
*HbERF-IVa2*			5.92	4.83	0.33	DREB2C	Heat stress tolerance	*Ricinus*	[51]				
*HbERF-IVa3*			2.85	2.52		DREB2B	Water deprivation stimulus	*Arabidopsis*	[56]	.			
*HbERF-Vb2*			3.24	7.39		RAP2.2	Response to hypoxic stress	*Arabidopsis*	[57]	.			
*HbERF-VI1*					3.03	CRF2		*Vitis*	[58]	CRF2/TMO3	At4g23750	Related to root initiation at later embryonic stages	[58,59]
*HbERF-VI2*	0.10	0.06				CRF2	Development of embryos and response to cytokin	*Arabidopsis*	[58]				
*HbERF-VI-L1*			5.81	4.97		ERF	Control of ethylene-responsive transcription genes	*Ricinus*	[60,61]	.			
*HbERF-VIIa1*					3.65	AP2/ERF	Response to biotic and abiotic stress conditions	*Populus*	[45]	.			
*HbERF-VIIa3*			4.23		7.07	AP2/ERF	Response to biotic and abiotic stress conditions	*Populus*	[45]	.			
*HbERF-VIIa4*	0.22	0.21	4.62		5.29	AP2/ERF	Response to biotic and abiotic stress conditions	*Populus*	[45]	.			
*HbERF-VIIa17*			49.10	9.81		ERF-2	Control of ethylene-responsive transcription genes	*Gossypium*	[60,61]	.			
*HbERF-VIIIa3*	0.15	0.10		017		ERF3	Control of ethylene-responsive transcription genes	*Arabidopsis*	[62]	.			
*HbERF-VIIIa7*			4.83	4.32		ATERF-4	Modulates ethylene and abscisic acid responses	*Arabidopsis*	[62]	.			
*HbERF-VIIIa11*					0.16	ATERF-4	Modulates ethylene and abscisic acid responses	*Arabidopsis*	[62]	.			
*HbERF-IXb2*			2.65	5.07		ERF	Control of ethylene-responsive transcription genes	*Olimarabidopsis*	[60,61]		At5g07580	Unknown	
*HbERF-IXb4*					0.17	ERF	Control of ethylene-responsive transcription genes	*Ricinus*	[60,61]	.			
*HbERF-IXb8*					0.07	ERF1	Drought, salt and freezing tolerances	*Malus*	[60,61]	.	At5g51190	Unknown	
*HbERF-IXc4*			57.10	10.50		ERF1	Drought, salt and freezing tolerances	*Ricinus*	[60,61]	.			
*HbERF-Xb1*					0.02	AP2/ERF	Response to biotic and abiotic stress conditions	*Populus*	[45]	RRTF1	At4g34410	Regulates redox homeostasis related to photosynthetic stress	[63]
*HbAP2-1*	0.14	0.11				ANT	Regulates up-regulation of genes establishing organ polarity and those specifying organ identity	*Ricinus*	[19,64]	BBM	At5g17430	Promotes cell proliferation and morphogenesis during embryogenesis	[18,65]
*HbAP2-3*			5.74	4.78		BBM	Cell proliferation and morphogenesis during embryogenesis	*Ricinus*	[18]	AIL7/PLT7	At5g65510	Regulates radial pattern formation process of a shoot apical meristem.	[66]
										AIL6/PLT3	At5g10510	Regulation of floral meristem growth	[67]
*HbAP2-5*	0.13	0.10				AP2/ERF	Response to biotic and abiotic stress conditions	*Ricinus*	[45]	.	At2g41710	Unknown	
*HbAP2-7*			24.90	12.90		AIL	Related to floral development	*Vitis*	[64]	ANT/DRG/CKC/CKC1	At4g37750	Regulates up—regulation of genes establishing organ polarity and those specifying organ identity	[19,64]
*HbAP2-12*	0.13	0.10				AP2	Meristem maintenance and cell differentiation	*Arabidopsis*	[68,69]	AP2/FLO2/FL1	At4g36920	Meristem maintenance and cell differentiation	[68,69]
*HbAP2-18*	0.22	0.16				AP2		*Vitis*					
*HbRAV-3*			43.60	16.40		RAV1	Leaf maturation and senescence	*Ricinus*	[70]	.			

The ratios between the relative transcript abundance of the regenerant and non-embryogenic lines (R/NE), the regenerant and embryogenic lines (R/E), of the normal and abnormal embryos (Nor/Ab) are presented for only significant down-regulated genes (< 1) and up-regulated (> 1).

The role of these gene expression markers was first predicted by a reciprocal best hit analysis (BLASTX) of the transcript sequences (Table 2). Roles were mostly found in the response to biotic and abiotic stress, and more precisely in tolerance of dehydration, salinity and cold. The genes of the AP2 and RAV families, along with a small proportion of ERFs, played a role in developmental processes (embryogenesis and flower development). In order to find *Hevea* potential orthologs to *Arabidopsis* genes functionally described in the literature, the deduced amino acid sequences of the AP2 domain were analysed for the AP2 family and the different ERF groups (I, III, IV, VI, X) making up the marker genes in *Hevea* (Additional file 1: Figure S1, Additional file 2: Figure S2, Additional file 3: Figure S3, Additional file 4: Figure S4, Additional file 5: Figure S5 and Additional file 6: Figure S6). The summary of the phylogenetic tree analyses can be found in Table 2. Thirteen of the *Hevea* marker genes proved to be potential orthologs of sixteen *Arabidopsis* genes including some co-orthologs: *HbERF-Ia1* with *ERF53*, *HbERF-IIIc1* with *AtCBF/DREB (CBF3/DREB1A, CBF2/DREB1C, CBF1/DREB1B, CBF4/DREB1D, DDF1, DDF2)*, *HbERF-IIIe3* with *TINY*, *HbERF-IVa1* and *HbERF-IVa2* with *DREB2A, HbERF-VI1* and *HbERF-VI2* with *CRF2/TMO3, HbERF-Xb1* with *RRTF1*, *HbAP2-1* with *BBM*, *HbAP2-3* with two *AINTEGUMENTA-Like* genes (*AIL7/PLT7* and *AIL6/PLT3*), *HbAP2-7* with *AINTEGUMENTA*, and *HbAP2-12* and *HbAP18* with *APETALA2.*

## Discussion

### Factors contributing to the loss of embryogenic capacity

Although callus browning occurs late during embryo development in the normal process, early browning in proliferating calli contributes to a loss of their embryogenic capacity by promoting the differentiation of active cells (meristematic and embryogenic) [25]. In *Hevea,* such browning has been linked to a strong accumulation of oxidized polyphenols in cells and to ethylene production [4,30,71]. This study corroborates those findings with the activation of ethylene biosynthesis and signalling genes in proliferating calli of low embryogenic or non-embryogenic lines (Figure 3), whereas this only occurred after induction in embryogenesis on EXP medium for the regenerant line. The gene expression markers of plant regeneration capacity found in proliferating calli belonged to groups VI, VII and VIII of the ERFs (*HbERF-VI2, HbERF-VIIa4, HbERF-VIIIa3*), which are factors of response to hormonal signals (ethylene, jasmonate, etc.). Other factors generally linked to development were also activated in proliferating calli from the embryogenic or non-embryogenic lines. Four genes from the AP2 family can be noted: *HbAP2-1, HbAP2-5, HbAP2-12, HbAP2-18*. The phylogenetic analysis between the *Hevea* and *Arabidopsis* families led to the prediction of several orthologs. *HbAP2-1* and *HbAP2-12/HbAP2-18* were found to be potential orthologs of *Arabidopsis BBM* and *AP2* genes, respectively [18,60]. *BBM* is preferentially expressed in developing embryos and seeds [18]. Its ectopic expression in *Arabidopsis*, *Brassica* and *Nicotiana* has led to the spontaneous formation of somatic embryos and cotyledon-like structures in seedlings [18,72]. However, such ectopic expression gives rise to pleiotropic phenotypes such as neoplastic growth, regeneration of plants on a hormone-free medium, and an alteration of leaf and flower morphology. The role of BBM in promoting cell proliferation and morphogenesis during embryogenesis seems to be confirmed for other species such as *Brassica napus* and *Elaeis guineensis*[17,73]. The *AP2* gene is involved in the control of *Arabidopsis* flower and seed development [68]. This gene is known to be expressed in non-floral organs, such as leaves and stems, and may play a general role in controlling *Arabidopsis* development. Nevertheless, early activation of these genes, especially *HbAP2-12* and *HbAP2-18*, in proliferating *Hevea* callus might be not appropriate since it is not conducive to further induction of somatic embryogenesis.

### Change occurring during somatic embryogenesis induction

Somatic embryogenesis is triggered by reducing the concentration of growth regulators in the culture medium of *Hevea* calli. This helps to slow down callus growth to the benefit of embryo formation [74]. Embryogenesis induction is also accompanied by callus browning. In this study, that transition was linked to changes in gene expression. Several ethylene biosynthesis genes, such as *HbACS3* and *HbACO2,* were highly transcribed in the calli before and after embryogenesis induction. When calli were transferred to the EXP medium, transcripts of the *SAMS* gene accumulated dramatically. That gene is also a marker that differentiated between the regenerant line and the other two low- or non-embryogenic lines, be it for the callus proliferation phase or during somatic embryogenesis induction. *SAMS* catalyses the formation of S-adenosyl methionine, which is a substrate for the ethylene and polyamine biosynthesis pathways. The latter would seem to play a decisive role in the somatic embryogenesis of *Hevea*[75]. However, the induction of several ERFs indicates the establishment of ethylene signalling. Four ERFs (*HbERF-IVa1*, *HbERF-Vb2*, *HbERF-Vb3*, *HbERF-VI2*) and the *HbRAV4* gene were transiently induced in calli on the EXP culture medium for somatic embryogenesis induction (Figure 6). At that stage of the process, the comparison between the regenerant line and the low- or non-embryogenic lines also revealed strong induction of 13 ERFs (*HbERF-IIIa1, HbERF-IIIb2, HbERF-IIIc1, HbERF-IIIe3, HbERF-IVa1, HbERF-IVa2, HbERF-IVa3, HbERF-Vb2, HbERF-VI-L1, HbERF-VIIa17, HbERF-VIIIa7, HbERF-IXb2, HbERF-IXc4*), 2 AP2 (*HbAP2-3, HbAP2-7*) and the *RAV3* gene (Figure 4 or Table 2).

Several of these gene expression markers were predicted to be orthologs of *Arabidopsis* genes with characterized functions. *HbERF-IIIc1* is orthologous to several *Arabidopsis DREB1s* from group 1 induced by cold [52,53]. *HbERF-IIIe3* is the putative ortholog of *TINY* known to be activated by drought, cold, ethylene and, to a lesser degree, methyl jasmonate [47]. The semi-dominant *tiny* mutation causes a reduction in plant height, and affects hypocotyl elongation and fertility. *TINY* might play a role in communication between biotic and abiotic stress signalling pathways. *HbERF-IVa1* and *HbERF-IVa2* are two potential orthologs of *DREB2A* involved in drought-responsive gene expression [50]. *HbERF-IVa1* was transitionally induced in calli placed on somatic embryogenesis induction medium, and both *HbERF-IVa1* and *HbERF-IVa2* were good markers of the regeneration potential compared with the low-embryogenic or non-embryogenic line. These two genes could therefore be very good gene expression markers but would also seem to play a key role in the somatic embryogenesis process. The transcripts of *HbERF-VI1* were accumulated in the normal embryos while *HbERF-VI2* showed under-expression in the proliferating calli of the regenerant line. These genes are putative orthologs to *CRF2*, which has been previously described as *TARGET OF MP3 (TMO3)*. The *TMO* gene is targeted by the auxin-dependent transcription factor MONOPTEROS (MP), which is a regulatory signal in embryonic root specification [59]*. AP2* genes are generally transcribed in multiple tissues during development. AILs also play a role in the specification of meristematic or division-competent states [76]. *HbAP2-3* is the putative ortholog of two *Arabidopsis* genes, *AIL7/PLT7*[66] and *AIL6/PLT3,* involved in floral meristem growth [67]. *HbAP2-7* would seem to be orthologous to the *ANT* gene [19]. ANT regulates cell proliferation and organ growth by maintaining the meristematic competence of cells during organogenesis [77]. More recently, it was shown to be promoting the initiation and growth of lateral organ primordia, and organ polarity [64]. Consequently, its very high expression in regenerant *Hevea* callus lines, compared with non-embryogenic and embryogenic lines, was in line with its role in embryo development. With regard to RAV, this family is regulated by ethylene [78] and brassinosteroids [79]. RAVs are involved in the response to biotic and abiotic stress [80]. The increase in *HbRAV4* transcripts during somatic embryogenesis induction is in accordance with dramatic changes provoked by ethylene.

### Control of development and of somatic embryo quality

The ontogenesis of somatic embryos involves an embryo growth phase, followed by the formation of apical meristem and roots, along with the procambial bundles, and lastly the accumulation of reserves needed for germination [81]. Somatic embryos gradually become dehydrated to acquire the quiescent state [82]. The analysis of *AP2/ERF* gene expression carried out at the end of the somatic embryo maturation phase revealed that the relative transcript abundance was very high for a large number of genes involved in ethylene biosynthesis and signalling, along with several *AP2* genes, including *HbAP2-3*, *HbAP2-6*, *HbAP2-9*, *HbAP2-10*, *HbAP2-13*, *HbAP2-18* and *HbAP2-20* (Figure 5). The potential roles previously described for HbAP2-3 (orthologous to AIL6 and AIL7) and for HbAP2-18 (orthologous to APETALA2) highlight the importance of these genes. All the 16 marker genes discriminating between the normal and abnormal embryos belonged to the ERF family. The abnormal embryos accumulated the transcripts of 6 ERFs belonging to group I (DREB subfamily [50]) more than the normal embryos did. The abundance of the transcripts of another DREB gene, HbERF-IVa2 orthologous to DREB2A, in the normal embryos is worth noting. This suggests differential regulation between the two types of embryos for the ERFs involved in the response to dehydration, salinity and cold.

## Conclusion

Of the 132 *AP2/ERF* genes studied, 40 were expression markers linked to the different stages of the somatic embryogenesis process in *Hevea*. With the identification of 11 very early markers, it was possible to predict the regeneration potential of proliferating callus lines, which opens up prospects for their application in selecting lines of interest for large-scale propagation. The phylogenetic analysis made it possible to predict more precisely the function of certain genes characterized already in *Arabidopsis*. The functions of 9 markers suggested that the regulation of hormone and stress signals play just as important a role in somatic embryogenesis as the genes involved in morphogenesis regulation. In addition to these marker genes, another *ERF* gene expressed during somatic embryogenesis is a potential ortholog of CRF (CYTOKININ RESPONSE FACTOR) encoded by *HbERF-VI1 or HbERF-VI2*[58]. An in-depth functional characterization of these markers should lead to a better understanding of somatic embryogenesis and explain the loss of embryogenic capacity, and embryo abnormality. Genetic variability in these genes could also be studied to determine whether allelic variations can be used in breeding programmes to select *Hevea* clones not only for agronomic traits but also for their responsiveness to somatic embryogenesis. The AP2/ERF superfamily could thus play a major role for several other biological functions in *Hevea*. Firstly, rubber production is stimulated by applying ethephon. In cases of over-tapping *in situ* coagulation of rubber particles leads to production losses: this is tapping panel dryness. Secondly, the *ERF1*, *ERF2*, *ERF3* and *RAV1* genes, corresponding to *HbERF-VIIa1*, *HbERF-VIIa3*, *HbERF-VIIa17* and *HbRAV1*[35], are induced at the same time as secondary laticifer differentiation [83]. Studying this superfamily in *Hevea* thus provides some new biological knowledge.

## Methods

### Plant material

The plant regeneration procedure from somatic embryogenesis consists of four steps described in Figure 1. Firstly, primary somatic embryogenesis was carried out using the inner integument of immature seeds from the *Hevea* clone PB 260 [24]. Secondly, friable callus lines were established from fragments of somatic embryos [29]. Thirdly, callus lines were cryopreserved for long-term storage and to avoid long-term subculturing [33,39]. Fourthly, embryo development and plant regeneration were induced from friable callus after thawing treatments [24].

In this study, three callus lines with different embryogenic capacity were previously identified from cryopreserved material [33]. The non-embryogenic callus line CI04115 does not have the ability to produce somatic embryos. The embryogenic but non-regenerant callus line CI04106 can produce a few somatic embryos but cannot regenerate plantlets. The regenerant callus line CI07060 can regenerate embryos and plantlets. These cryopreserved callus lines were thawed by immersing the cryovials in a warm water-bath maintained at 37°C for 2 min. ENT medium was prepared with the basic MH macro and microelements supplemented with 9 mM CaCl_2,_ 234 mM sucrose, 30 μM AgNO_3_, 1.34 μM BAP, 1.34 μM 3,4-D, 0.5 μM ABA and 2.3 g.L^-1^ Phytagel [24]. The content of the cryovials was placed for 1 h in Petri dishes containing 25 mL of ENT post-thawing medium, which is a modified ENT medium with 1 M sucrose and 1 mM CaCl_2_[33]. Calli were then transferred for 1 day to fresh ENT post-thawing 2 medium supplemented with 0.5 M sucrose, after which they were transferred again to ENT recovery medium containing a normal sucrose concentration (234 mM) and 1 mM CaCl_2_. After callus growth recovery in Petri dishes, friable callus aggregates were isolated and transferred to test tubes containing ENT medium. Calli were grown in the dark at 27°C and subcultured regularly every two weeks on fresh ENT medium.

For embryo induction, one gram of friable callus proliferating on ENT medium was transferred to a 250-mL bottle containing 50 mL of expression medium (EXP). EXP was a modified ENT medium supplemented with 9 mM CaCl_2,_ 58.5 mM sucrose, 175.5 mM maltose, 0.44 μM BAP, 0.44 μM 3,4-D and 0.5 μM ABA. This culture was incubated in the dark at 27°C for 4 weeks. Development of somatic embryos was obtained by transferring callus from one bottle to a temporary immersion system containing 200 mL of liquid DEV medium (RITA®, CIRAD, France). There were two successive subcultures of 4 weeks with 1 min of immersion per day in DEV medium, which had the same composition. The DEV medium was a modified ENT medium supplemented with 3 mM CaCl_2_ and without plant growth regulators. After 8 weeks of culture, mature embryos with a well-formed embryonic axis and cotyledons (normal) were transferred to glass tubes containing a germination medium (GER), which contained MS macro-elements, MH micro-elements and vitamins, and 234 mM sucrose, semi-solidified with 7 g/L Agar. Embryos were cultured under a light intensity of 60 μmol m^-2^ s^-1^ with a 12 h day/12 h dark photoperiod for embryo conversion into plantlets. Abnormal embryos were counted and discarded at the end of DEV2.

### Total RNA isolation

For the callus lines with different embryogenic capacities, callus was collected at the end of the ENT and EXP cultures. For the regenerant callus line, normal and abnormal embryos were collected at the end of DEV2. Leaves, stems and roots were collected from one-month-old plantlets. All samples were frozen in liquid nitrogen and stored in the freezer at -80°C pending total RNA extraction. Total RNAs were isolated using the caesium chloride cushion method adapted from Sambrook and coll. [84] by Duan and coll. [38]. One gram of fresh matter was ground and transferred to a tube containing 30 mL of extraction buffer consisting of 4 M guanidium isothiocyanate, 1% sarcosine, 1% polyvinylpyrrolidone and 1% ß-mercapto-ethanol. After homogenization, tubes were kept on ice and then centrifuged at 10,000 g at 4°C for 30 min. The supernatant was transferred to a new tube containing 8 mL of 5.7 M CsCl. Ultracentrifugation in a swinging bucket was carried out at 89,705 g at 20°C for 20 h. The supernatant and caesium cushion were discarded whilst the RNA pellet was washed with 70% ethanol. After 30 min of air drying, the pellet was dissolved in 200 μL of sterile water. Although DNA could not cross the caesium cushion for this centrifugation condition, DNA contamination was checked by PCR amplification using primers of the Actin gene including the intron sequence. RNAs were conserved at -80°C.

### Primer design and analysis of transcript abundances by real-time RT-PCR

Several rules were applied in order to reduce the risk of error in relative gene expression data. The integrity of total RNA was checked by electrophoresis. Primers were designed at the 3’ side of each sequence in order to reduce the risk of error due to short cDNA synthesis using the Primer 3 module of Geneious (Biomatters Ltd., New Zealand). Real-time PCR amplification and the fusion curve were carried out using a mix of cDNAs in order to check the specificity of each pair of primers. Primer sequences are listed in Additional file 7: Table S1.

cDNAs were synthesized from 2 μg of total RNA to the final 20 μL reaction mixture using a RevertAidTM M-MuLV Reverse Transcriptase (RT) kit according to the manufacturer's instructions (MBI, Fermentas, Canada). Full-length cDNA synthesis was checked on each cDNA sample by PCR amplification of the Actin cDNA using primers at the cDNA ends. Quantitative gene expression analysis was finally carried out by real-time RT-PCR using a Light Cycler 480 (Roche, Switzerland). Real-time PCR reaction mixtures consisted of 2 μL RT product cDNA, 0.6 μL of 5 μM of each primer, and 3 μL 2 × SYBR green PCR master mix (LightCycler® 480 SYBR Green I Master, Roche Applied Sciences) in a 6-μL volume. PCR cycling conditions comprised one denaturation cycle at 95°C for 5 min, followed by 45 amplification cycles (95°C for 20 s, 60°C for 15 s, and 72°C for 20s). Expression analysis was performed in a 384-well plate. Samples were loaded using an automation workstation (Biomek NX, Beckman Coulter).

Real-time PCR was carried out for eleven housekeeping genes in order to select the most stable gene as the internal control for all the compared tissues (HbelF1Aa, HbUBC4, HbUBC2b, HbYLS8, HbRH2b, HbRH8, HbUBC2a, HbalphaTub, Hb40S, HbUbi, HbActin) (Additional file 8: Table S2). *HbRH2b* was selected as the best reference gene due to its stability in tissues from various stages of somatic embryogenesis. The homogeneity of the *HbRH2b* gene Cp confirmed that it could be used as an internal reference gene (Table 3). The *HbRH2b* gene was amplified in each reaction plate in parallel with target genes. The transcript abundance level for each gene was relatively quantified by normalization with the transcript abundance of the reference *HbRH2b* gene. Relative transcript abundance took into account primer efficiencies. All the normalized ratios corresponding to transcript accumulation were calculated automatically by Light Cycler Software version 1.5.0 provided by the manufacturer using the following calculation: Normalized Ratio = Efficiency ^-Δ*(Cp target-Cp RH2b)*^*.*

**Table 3:** Comparison of Cp values, standard deviation and coefficient of variance for gene expression analysis by real-time RT-PCR of 11 housekeeping genes in 11 tissues from various stages of somatic embryogenesis

**Gene**	**Cp Mean**	**Standard deviation**	**Coefficient of variance**
HbelF1Aa	23.64	1.22	0.052
HbUBC4	28.31	2.62	0.093
HbUBC2b	21.70	1.73	0.08
HbYLS8	23.23	1.24	0.053
HbRH2b	22.69	1.01	0.045
HbRH8	25.04	1.21	0.048
HbUBC2a	24.93	1.40	0.056
HbalphaTUB	26.87	1.23	0.046
Hb40S	29.12	1.92	0.066
HbUbi	33.56	1.61	0.048
HbActin	22.88	1.47	0.064

### Statistical data analyses

Each callus line was maintained in three biological replicates. Morphological data were recorded and calculated per gram of callus transferred to EXP medium. Statistical analysis was performed with an ANOVA followed by a Student Newman-Keuls test. Values with the same letter did not differ significantly at the 0.05 probability level in Table 1.

Real-time PCR reactions were set up with three biological replications. Statistical analysis was performed with an ANOVA after logarithmic transformation of raw data. The ANOVA was followed by a Student Newman-Keuls test when values of relative transcript abundances were compared for each stage of the somatic embryogenesis process between the three callus lines with different embryogenic capacity (regenerant, embryogenic and non-embryogenic lines), the two types of embryos (normal and abnormal), and the tissues from the normal somatic embryogenesis process from callus to plantlets (regenerant callus on ENT and EXP media, normal embryo, leaf, stem and root). Values with the same letter did not differ significantly at the 0.05 probability level.

In Table 2, the level of expression was calculated as the ratio between the relative transcript abundances in callus of the regenerant line/embryogenic line, the regenerant line/non-embryogenic line grown on ENT and EXP media, and in normal/abnormal somatic embryos. It was considered as an up-regulation with a ratio > 1.0, and a down-regulation with a ratio < 1.0. The statistical analysis was carried out from the logarithm of raw data using the two-tailed probability values of the *t* test. The ratio of the relative transcript abundances with a p-value ≤ 0.05 was adopted as significant for down or up-regulation. Only significant data are discussed in the manuscript.

### Phylogenetic analysis of the AP2 domain from AP2/ERF marker genes

A multiple alignment analysis was performed on full-length AP2 domain sequences from *Hevea* and *Arabidopsis* for the AP2, ERF (groups III, IV, V, VII, VIII, X) and RAV families (Additional file 1: Figure S1, Additional file 2: Figure S2, Additional file 3: Figure S3, Additional file 4: Figure S4, Additional file 5: figure S5 and Additional file 6: Figure S6). The full AP2-domain sequences derived from *Hevea* and *Arabidopsis* AP2-domain proteins of around 60 amino acids were then aligned using MUSCLE software [85,86], which uses a progressive multiple alignment method. The alignment was curated by Gblocks software [87], searching for at least 10-amino-acid-long conserved blocks, and the block with 57 amino acids was extracted. This block of 57 amino acids was used to construct the phylogenetic tree using PhyML software [88], which implements a maximum likelihood tree reconstruction method, using the LG+gamma model, starting from a BioNJ tree [89]. A RAP-Green analysis was performed from the initial PhyML tree to improve gene function inferences and predict gene duplications in phylogenetic trees [90]. The final tree was drawn and displayed with the Archaeoptheryx program, and rooted on the branch separating the RAV family from the rest of the tree. Branch supports were computed using the aLRT-SHlike method [91].

## Authors’ contributions

FD and FM established the various friable callus lines for this experiment from cryopreserved material. PP induced somatic embryogenesis and regenerated both embryos and plantlets, and then carried out the total RNA isolation and cDNA synthesis from samples. PP and RP designed the primers from the AP2/ERF genes. MR and JL supervised the real-time RT-PCR analysis on the Roche platform. LL supervised the tissue culture experiments. JFD advised on the strategy and supervised the phylogenetic analyses. PP and PM drafted the manuscript. PM coordinated this research work. All the authors read and approved the final manuscript.

## Supplementary Material

Additional file 1: Figure S1 Phylogenetic tree of the AP2 family. The deduced amino acid sequences of the AP2 domain from *Hevea* (black letter) and *Arabidopsis* (blue letter) were aligned using Muscle, and the phylogenetic tree was constructed using PhyML with an LG+T model. *Hevea* somatic embryogenesis marker genes are indicated in bold letters. Click here for file

Additional file 2: Figure S2 Phylogenetic tree of ERF group I. The deduced amino acid sequences of the AP2 domain from *Hevea* (black letter) and *Arabidopsis* (blue letter) were aligned using Muscle, and the phylogenetic tree was constructed using PhyML with an LG+T model. *Hevea* somatic embryogenesis marker genes are indicated in bold letters. Click here for file

Additional file 3: Figure S3 Phylogenetic tree of ERF group III. The deduced amino acid sequences of the AP2 domain from *Hevea* (black letter) and *Arabidopsis* (blue letter) were aligned using Muscle, and the phylogenetic tree was constructed using PhyML with an LG+T model. *Hevea* somatic embryogenesis marker genes are indicated in bold letters. Click here for file

Additional file 4: Figure S4 Phylogenetic tree of ERF group IV. The deduced amino acid sequences of the AP2 domain from *Hevea* (black letter) and *Arabidopsis* (blue letter) were aligned using Muscle, and the phylogenetic tree was constructed using PhyML with an LG+T model. *Hevea* somatic embryogenesis marker genes are indicated in bold letters. Click here for file

Additional file 5: Figure S5 Phylogenetic tree of ERF group VI. The deduced amino acid sequences of the AP2 domain from *Hevea* (black letter) and *Arabidopsis* (blue letter) were aligned using Muscle, and the phylogenetic tree was constructed using PhyML with an LG+T model. *Hevea* somatic embryogenesis marker genes are indicated in bold letters. Click here for file

Additional file 6: Figure S6 Phylogenetic tree of ERF group X. The deduced amino acid sequences of the AP2 domain from *Hevea* (black letter) and *Arabidopsis* (blue letter) were aligned using Muscle, and the phylogenetic tree was constructed using PhyML with an LG+T model. *Hevea* somatic embryogenesis marker genes are indicated in bold letters. Click here for file

Additional file 7: Table S1 List of primer sequences for 132 AP2/ERF genes from H. brasiliensis clone PB 260. Click here for file

Additional file 8: Table S2 Comparison Cp value of callus with various embryogenic capacities grown on embryogenesis induction medium (ENT) and somatic embryogenesis expression medium (EXP){Calli are from three types of lines: (NE) non-embryogenic line (CI04115), (E) embryogenic and non-regenerant line (CI04106), and (R) regenerant line (CI07060)}, normal and abnormal embryo and 3 organ of plantlet:(L) Leaf, (S) Stem, (R) Root in 11 house keeping genes. Click here for file
