# Supplementary figures and images for: Some ethylene biosynthesis and AP2/ERF genes reveal a specific pattern of expression during somatic embryogenesis in Hevea brasiliensis
Source: BMC Plant Biol. 2012 Dec 26;12:244. doi: 10.1186/1471-2229-12-244 (PMC3561283; doi:10.1186/1471-2229-12-244)

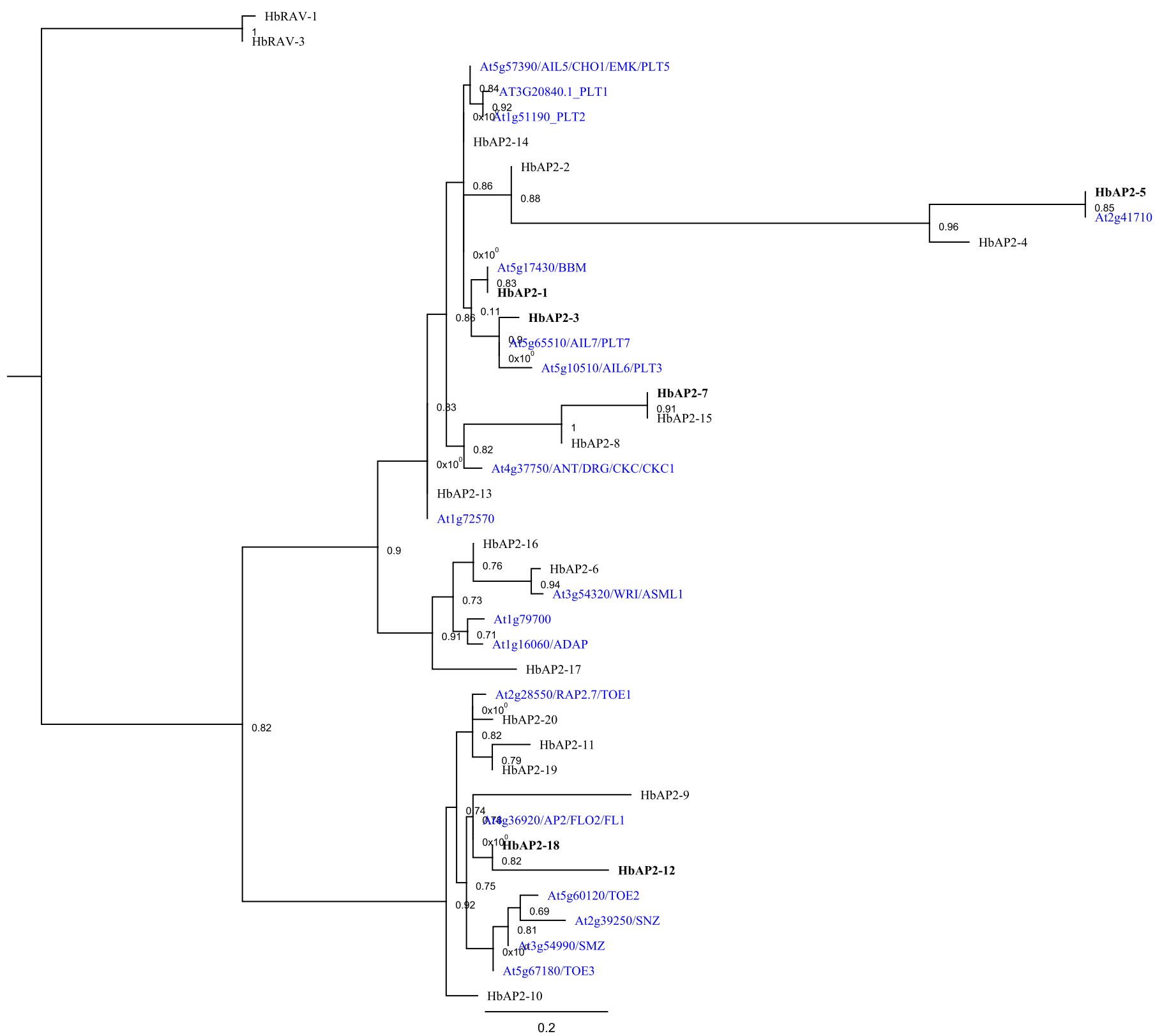

Supplement: Additional file 1: Figure S1 — Phylogenetic tree of the AP2 family. The deduced amino acid sequences of the AP2 domain from Hevea (black letter) and Arabidopsis (blue letter) were aligned using Muscle, and the phylogenetic tree was constructed using PhyML with an LG+T model. Hevea somatic embryogenesis marker genes are indicated in bold letters. [file 1471-2229-12-244-S1.pdf]

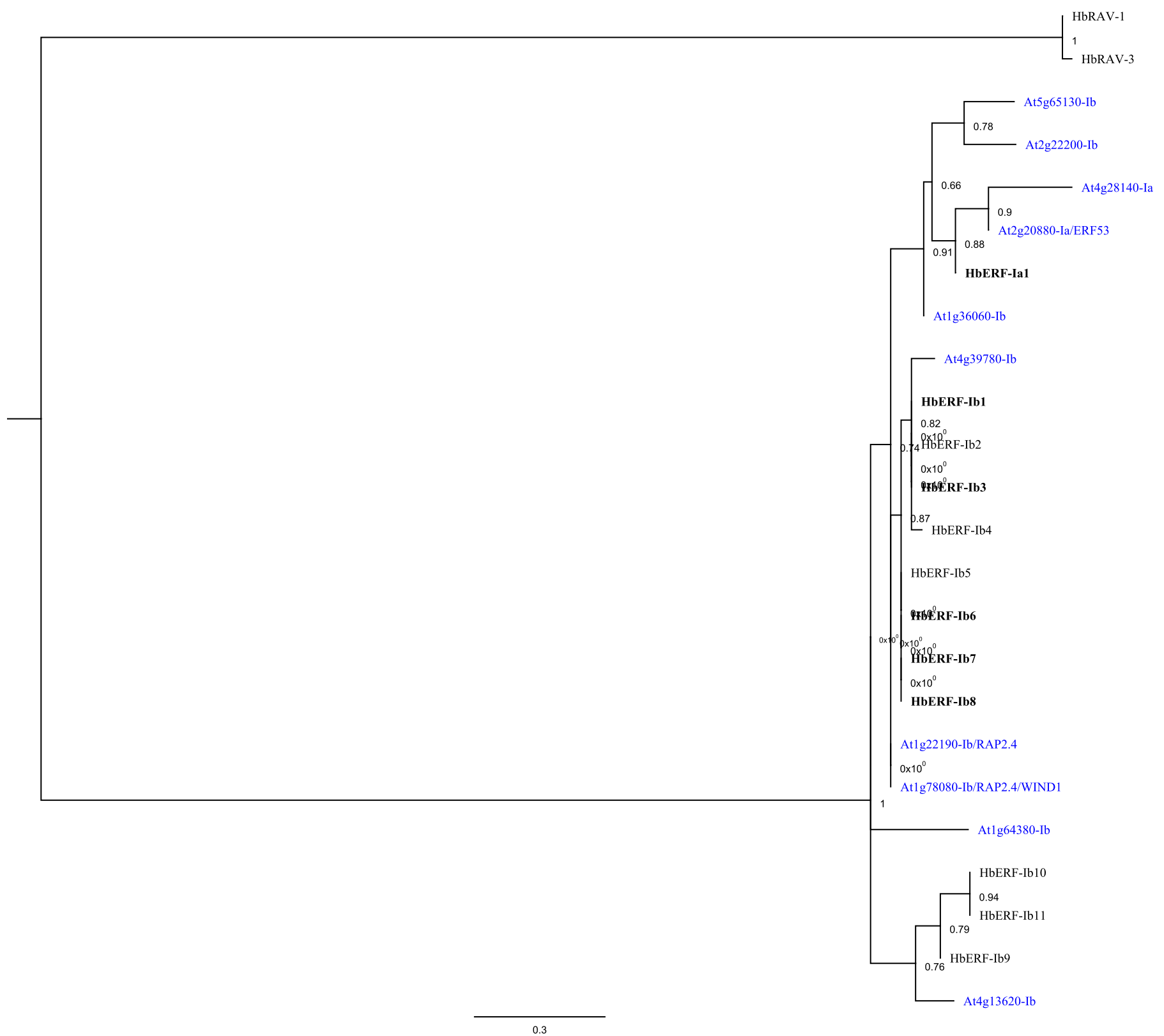

Supplement: Additional file 2: Figure S2 — Phylogenetic tree of ERF group I. The deduced amino acid sequences of the AP2 domain from Hevea (black letter) and Arabidopsis (blue letter) were aligned using Muscle, and the phylogenetic tree was constructed using PhyML with an LG+T model. Hevea somatic embryogenesis marker genes are indicated in bold letters. [file 1471-2229-12-244-S2.pdf]

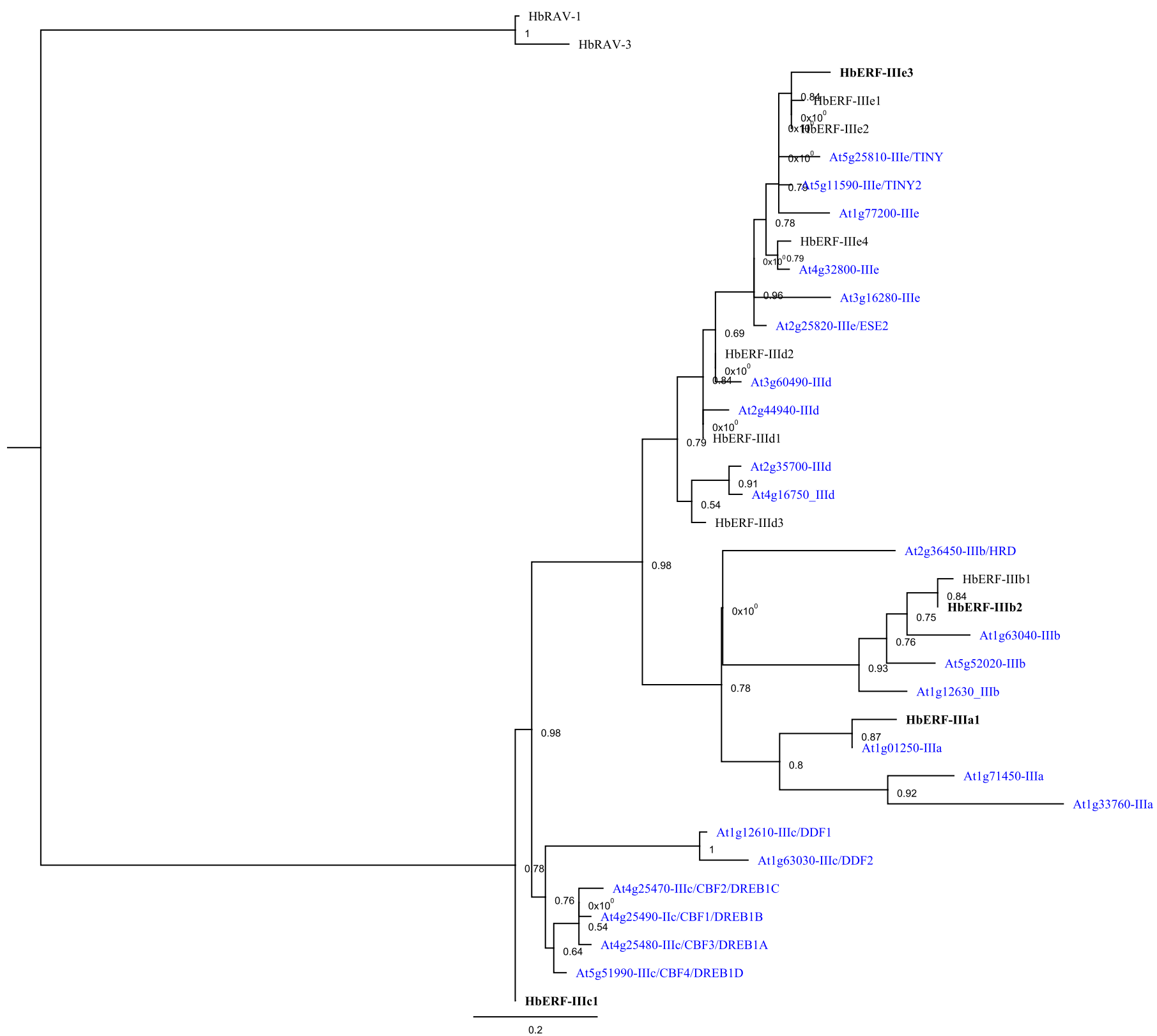

Supplement: Additional file 3: Figure S3 — Phylogenetic tree of ERF group III. The deduced amino acid sequences of the AP2 domain from Hevea (black letter) and Arabidopsis (blue letter) were aligned using Muscle, and the phylogenetic tree was constructed using PhyML with an LG+T model. Hevea somatic embryogenesis marker genes are indicated in bold letters. [file 1471-2229-12-244-S3.pdf]

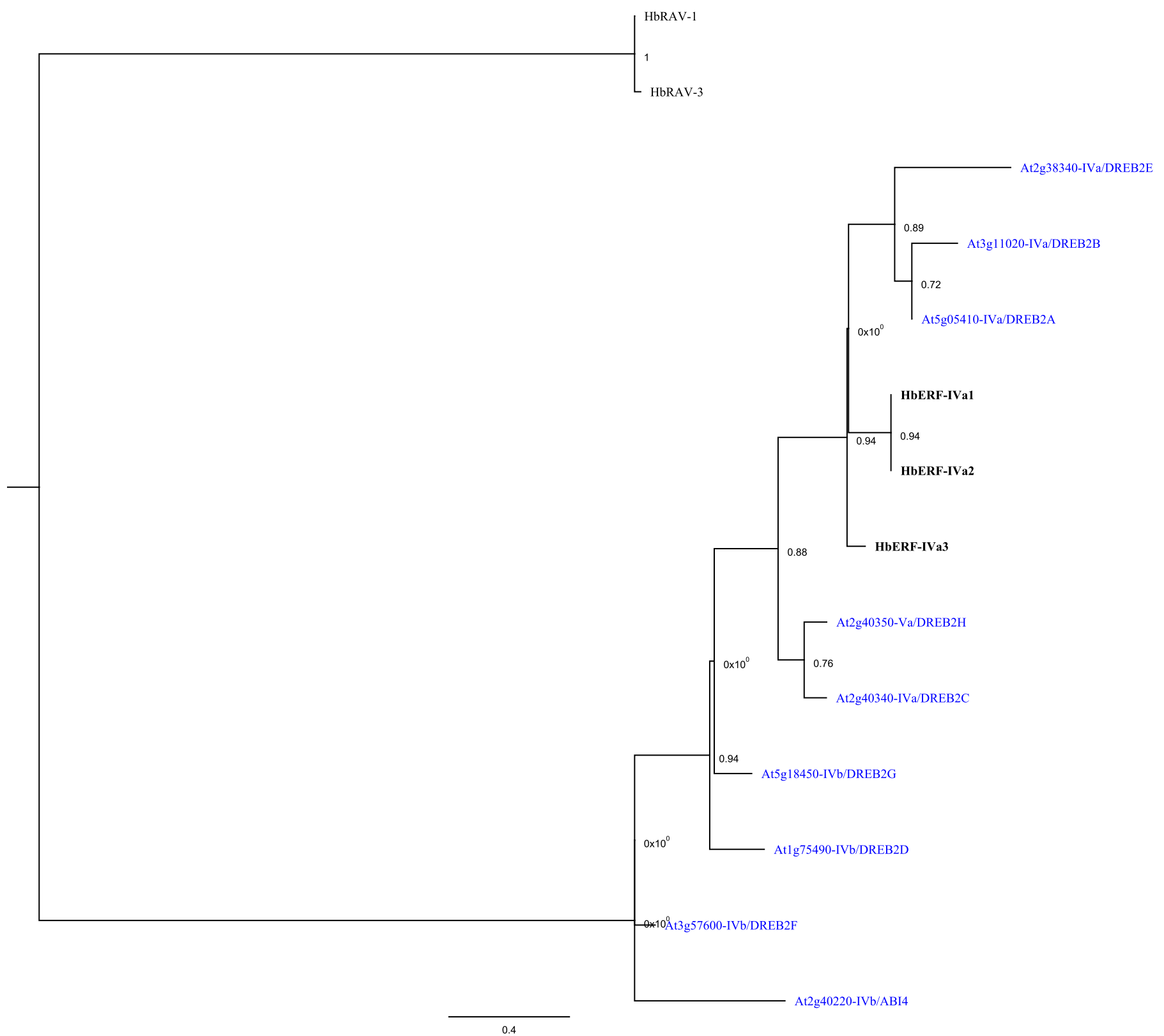

Supplement: Additional file 4: Figure S4 — Phylogenetic tree of ERF group IV. The deduced amino acid sequences of the AP2 domain from Hevea (black letter) and Arabidopsis (blue letter) were aligned using Muscle, and the phylogenetic tree was constructed using PhyML with an LG+T model. Hevea somatic embryogenesis marker genes are indicated in bold letters. [file 1471-2229-12-244-S4.pdf]

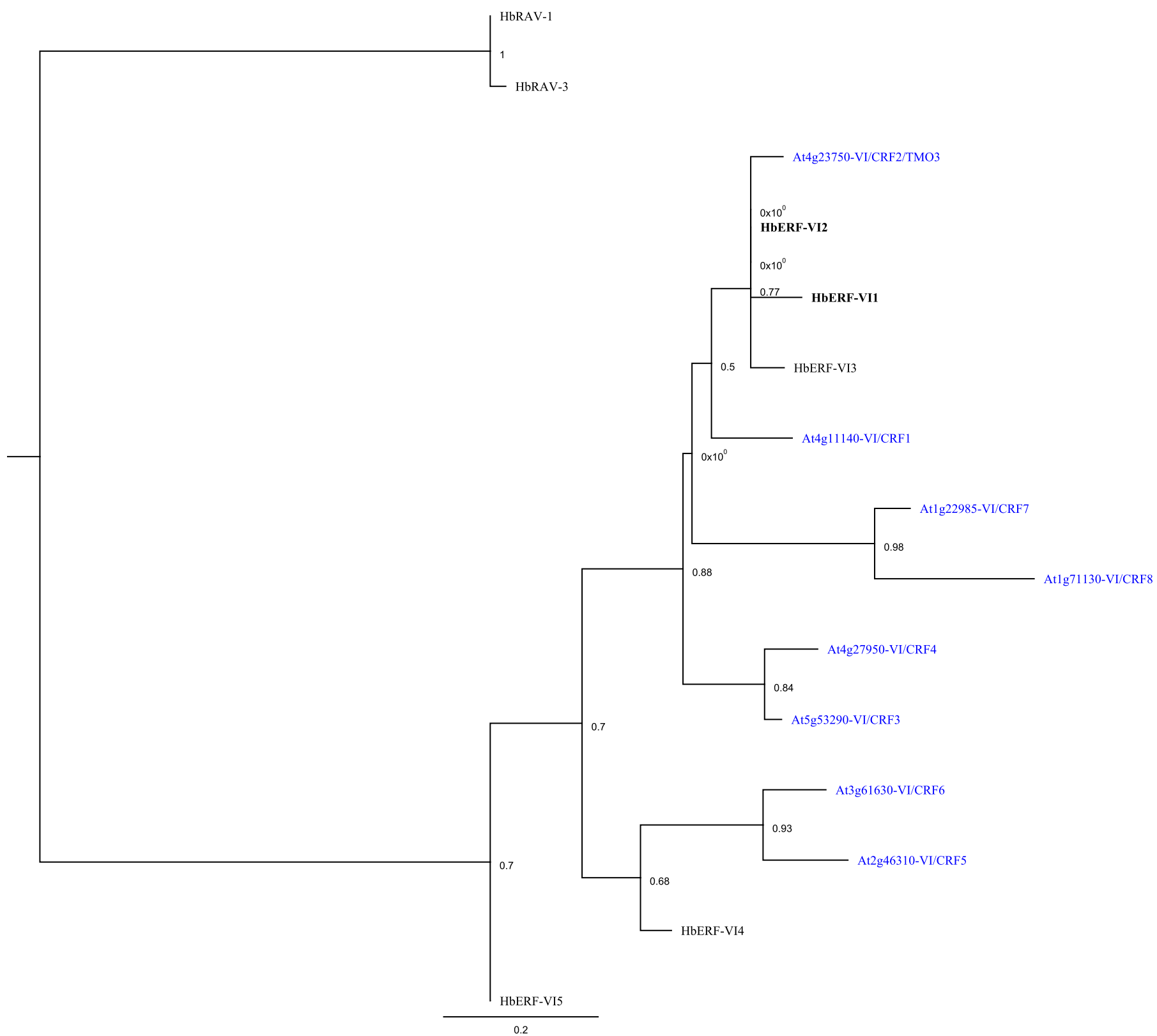

Supplement: Additional file 5: Figure S5 — Phylogenetic tree of ERF group VI. The deduced amino acid sequences of the AP2 domain from Hevea (black letter) and Arabidopsis (blue letter) were aligned using Muscle, and the phylogenetic tree was constructed using PhyML with an LG+T model. Hevea somatic embryogenesis marker genes are indicated in bold letters. [file 1471-2229-12-244-S5.pdf]

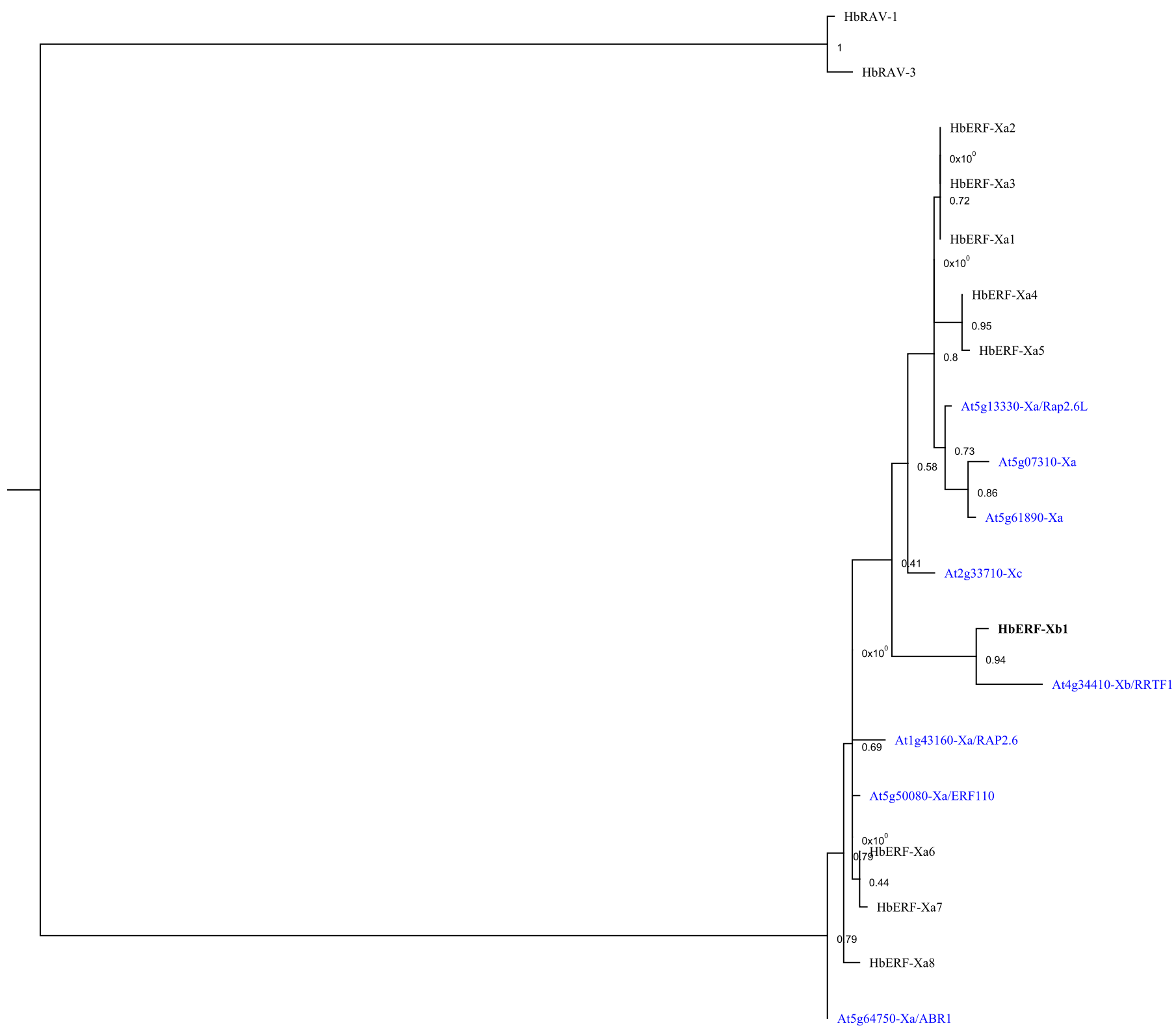

Supplement: Additional file 6: Figure S6 — Phylogenetic tree of ERF group X. The deduced amino acid sequences of the AP2 domain from Hevea (black letter) and Arabidopsis (blue letter) were aligned using Muscle, and the phylogenetic tree was constructed using PhyML with an LG+T model. Hevea somatic embryogenesis marker genes are indicated in bold letters. [file 1471-2229-12-244-S6.pdf]
